# Severe ROP rate and assessment of the burden of ROP screening at a single tertiary care public hospital in Pakistan

**DOI:** 10.1186/s12886-025-04437-6

**Published:** 2025-10-23

**Authors:** Muhammad Moin, Lubna Siddiq Mian, Miranda Martinez-Hussain, Muhammad Shahid, Clare Gilbert, Umar K. Mian

**Affiliations:** 1 King Edward Medical University, College of Ophthalmology and Allied Vision Sciences (COAVS), Mayo Hospital, Lahore, Pakistan; 2 Albert Einstein College of Medicine, Bronx, NY USA; 3 Ameer ud Din Medical College, Lahore General Hospital, Lahore, Pakistan; 4 Department of Clinical Research, International Centre for Eye Health, London School of Hygiene and Tropical Medicine, London, UK; 5 Departments of Ophthalmology and Pediatrics, Montefiore Medical Center/Albert Einstein College of Medicine, Bronx, NY USA

**Keywords:** Retinopathy of prematurity, ROP screening, Birth weight, Gestational age, ROP screening effort

## Abstract

**Background:**

To assess the rate of severe ROP and the burden of examining preterm infants requiring ROP screening.

**Methods:**

A prospective study of all inborn preterm infants eligible for ROP screening admitted to Lahore General Hospital (LGH) between 2015 and 2021 with a gestational age (GA) of ≤ 35 weeks or a birthweight (BW) of ≤ 2000 g. All infants had a dilated fundus exam, and the number of examinations and level of ROP were recorded.

**Results:**

3,521 infants met the screening criteria, 1,641 (46.7%) of whom were screened at least once. Among the 1880 (53.3%) not screened, 42.2% of eligible babies died and 11.2% were discharged and did not return for screening. Rates of any ROP, Type 1 ROP and more advanced ROP (stages 4 and 5) were 32.2%, 9.7% and 0.7%, respectively. Among the 170 infants with Type 1 and more advanced ROP (i.e. severe ROP) 45% had a GA of 30–35 weeks, 18.8% had a GA of ≥ 32 weeks, and 25% had a BW of > 1500 g. The average inpatient stay was 10.7 days, and nearly all infants required outpatient screening. The total number of examinations was 4,007 with an average of 2.4 per patient.

**Discussion:**

The third epidemic of ROP has arrived in Pakistan. There is an urgent need to scale up screening and treatment. Effective screening programs in public hospitals can be established with current resources. Initial screening guidelines need to include at least a GA of ≤ 35 weeks. Most infants will require outpatient screening after discharge. Estimates of screening and treatment burden can be made from these data for similar institutions initiating ROP screening programs.

**Conclusions:**

Effective screening is achievable in public hospitals using existing resources, especially with committed staff. The < 35 weeks GA protocol is essential, and outpatient care must be integrated into program planning. Limitations include single-site data and lack of retinal imaging, but all examinations were conducted by experienced ophthalmologists.

## Introduction

Retinopathy of prematurity (ROP) is a major cause of blindness in children in low- and middle-income countries (LMIC) [1]. The pathogenesis of ROP has been well described and involves oxygen-regulated and non-oxygen-regulated factors [2, 3]. Each year, around 32,300 infants globally become irreversibly blind or vision impaired from ROP [4]. 

Three epidemics of blindness from ROP have been described [5]. In the 1940 s and 1950 s, the first epidemic coincided with the widespread use of inadequately monitored or unmonitored 100% supplemental oxygen. The introduction of stricter oxygen monitoring and guidelines significantly reduced blindness. However, a second epidemic emerged in the 1970 s and 1980 s, as advances in neonatal care increased the survival of extremely premature infants who were at higher risk for ROP. A third epidemic is currently occurring in LMICs like Pakistan, where neonatal care has advanced enough to increase the survival of premature infants at a time when optimal ROP detection and treatment practices are not widely or uniformly implemented, and where neonatal care can be suboptimal leading to greater exposure to modifiable risk factors, including hyperoxia [6]. In these settings, more mature, larger preterm infants also develop treatment-requiring Type 1 ROP, which means that wider birthweight (BW) and gestational age (GA) criteria are needed than in high-income settings [7]. Indeed, our group reported that 51 children who presented blind from untreated ROP in Pakistan between 2017 and 2019 had a mean GA of 28.8 (26–38) weeks, and a mean BW of 1229 (800–2100)g [8]. 

Although there are no established national screening criteria for ROP in Pakistan due in part to a lack of data, the Ophthalmological Society of Pakistan (OSP) in 2019 suggested initial criteria of a GA of ≤ 35 weeks or a BW of ≤ 2000 g [9]. Two studies from private, level 3 NICUs in Pakistan suggest that infants weighing ≤ 1500 g or with a GA of ≤ 32 weeks are at risk for severe ROP in these settings [10, 11]. One public hospital using the criteria of GA ≤ 36 weeks or BW ≤ 3000 g (mean BW and GA: 1721 g and 32.43 weeks) reported any ROP in 15% and Type 1 ROP (defined as zone I, any stage ROP with plus disease; zone I, stage 3 ROP with or without plus disease; zone II, stages 2 or 3 ROP with plus disease) in 2.3% of the infants screened [12]. Another public hospital using the criteria GA ≤ 35 weeks and BW ≤ 2000 g (mean BW and GA: 1218.5 g and 28.6 weeks) reported any ROP in 27% and severe ROP in 5.8% of infants [13]. 

The aim of this study is to describe the rate of ROP, the screening load, and the burden of treatment-requiring ROP in premature infants with a GA of ≤ 35 weeks or a BW of ≤ 2000 g who received neonatal care in a public (government) hospital in Pakistan.

## Methods

### Study design

This study was a prospective case series, conducted between 2015 and 2021 at Lahore General Hospital (LGH) in Lahore, Pakistan. Data were collected by the ROP coordinator.

### Cohort

Inclusion criteria were all inborn, premature infants admitted to the NICU with a GA of ≤ 35 weeks or a BW of ≤ 2000 g. The ROP coordinator was responsible for identifying admitted infants who were eligible for screening. If they met the criteria, they were added to a list with the date of the first examination, approximately four weeks after birth. ROP screening was performed by two ophthalmologists using dilated fundus examination by indirect ophthalmoscopy with a 20-diopter lens. Signs of ROP were classified using the International Classification of ROP [14]. Cessation of ROP screening followed UK ROP guidelines [15]. If the infant was an inpatient, the examination was undertaken in the NICU. At discharge, an appointment was made for a follow-up examination in the eye department, if required. Contact telephone numbers were recorded, and the ROP coordinator called the parents/carers before the scheduled appointment. If the infant did not attend the screening, a follow-up phone call was made.

Severe ROP included Type 1 ROP (any ROP with plus disease in zone I, stage 3 ROP in zone I, or stage 2 or 3 ROP with plus disease in zone I) [16] and stages 4 and 5. After Anti-VEGF treatment infants were followed to a postmenstrual age (PMA) of 65 weeks or until ROP had fully regressed.

Infants were categorized into four groups by GA: Group A (GA ≤ 30 weeks), Group B (GA 30–32 weeks), Group C (GA 32+–35 weeks), and Group D (GA > 35 weeks), and three BW groups: Group 1 (≤ 1500 g), Group 2 (1501–2000 g), and Group 3 (> 2000 g).

## Results

### Study population

Between 2015 and 2021, a total of 3,523 infants met the inclusion criteria and were eligible for screening. 1,641 (46.6%) were screened at least once. The other 1,882 ((53.4%) were not screened on account of death, which occurred in 42.2% of eligible babies (inpatient mortality 35.4%; outpatient mortality 6.8%). A further 397 infants (11.2%) were not screened because they were lost to follow up after being discharged home before screening.

### Number of examinations

The mean GA of the infants screened was 32 (25–37) weeks and the mean BW was 1628 (600–2800)g. The 1,641 infants underwent a total number of 4,007 examinations, average 2.4 per infant. The total number of examinations and the average number of examinations declined with increasing GA (3.1, 2.7, 2, and 1.4 for groups A, B, C, and D, respectively), and with increasing BW (3, 2.2, and 1.9 for groups 1, 2, and 3, respectively) (Table 1).

**Table 1:** Screening examinations per infant and length of inpatient stay, by gestational age group and birthweight

Gestational age	Number of infants	Number of Exams	Examinations per infant	Inpatient days (SD, range)
≤ 30 weeks (A)	483	1432	3.1	12 ± 8 (3–59)
30 to < 32 weeks (B)	558	1415	2.7	11 ± 7 (3–60)
32 to 35 weeks (C)	591	1143	2.0	9 ± 6 (3–54)
> 35 weeks (D)	9	17	1.4	9 ± 3 (4–13)
Birthweight				
≤ 1500 g (1)	719	2048	3.0	13 ± 8 (3–60)
1501–2000 g (2)	766	1669	2.2	9 ± 6 (3–64)
> 2000 g (3)	156	290	1.9	9 ± 7 (3–50)

### Mean inpatient stay

The mean inpatient stay was 12.9 days which declined with increasing GA (10.6, 9.1, and 8.9 days for groups A, B, C, and D, respectively) and increasing BW (12.8, 9.2, and 9.1 days for groups 1, 2, and 3, respectively)(Table 1). Most infants had an inpatient stay of less than four weeks and would have been discharged before their first eye examination. 95% of even the most preterm infants (GA ≤ 30 weeks) had inpatient stays of less than four weeks.

### Rates of ROP

Overall, 520 (32.2%) infants developed ROP of any severity (Table 2). 358 (21.8%) infants developed mild ROP not requiring treatment and 159 (9.7%) developed Type 1 ROP. Eleven (0.7%) infants developed advanced ROP (stage 4 A, 3; stage 4B, 4 and stage 5, 4) all of whom were screened 3–4 weeks late. None of the infants who were screened on time developed Stage 4 or worse disease.

Rates of all severity of ROP declined with increasing GA and BW (Table 2). One infant with a GA > 35 weeks and two with a BW > 2000 g developed Type 1 ROP. All 11 infants with stage 4 and 5 ROP had a GA of < 35 weeks or a BW of < 2000 g.

**Table 2:** Severity of ROP, by gestational age and birthweight

Gestational age (weeks)		No ROP		Mild ROP		Type 1 ROP		Stage 4/5 ROP		Any ROP	
	*N*	%	%	*n*	%	*n*	%	*n*	%	*n*	%
≤ 30	483	215	44.5%	176	36.4%	88	18.2%	4	0.8%	268	16.3%
30 to < 32	558	399	71.5%	113	20.3%	45	8.1%	1	0.2%	159	9.7%
32 to 35	591	492	83.2%	68	11.5%	25	4.2%	6	1.0%	99	6.0%
> 35	9	7	77.8%	1	11.1%	1	11.1%	0	0.0%	2	0.1%
Total	1641	1113	67.8%	358	21.8%	159	9.7%	11	0.7%	528	32.2%
Birthweight											
≤ 1500 g	719	368	51.2%	227	31.6%	116	16.1%	8	1.1%	351	48.8%
1501–2000 g	766	603	78.7%	119	15.5%	41	5.4%	3	0.4%	163	22.7%
> 2000 g	156	142	91.0%	12	7.7%	2	1.3%	0	0.0%	14	1.9%
Total	1641	1113	67.8%	358	21.8%	159	9.7%	11	0.7%	528	32.2%

**Fig. 1:**
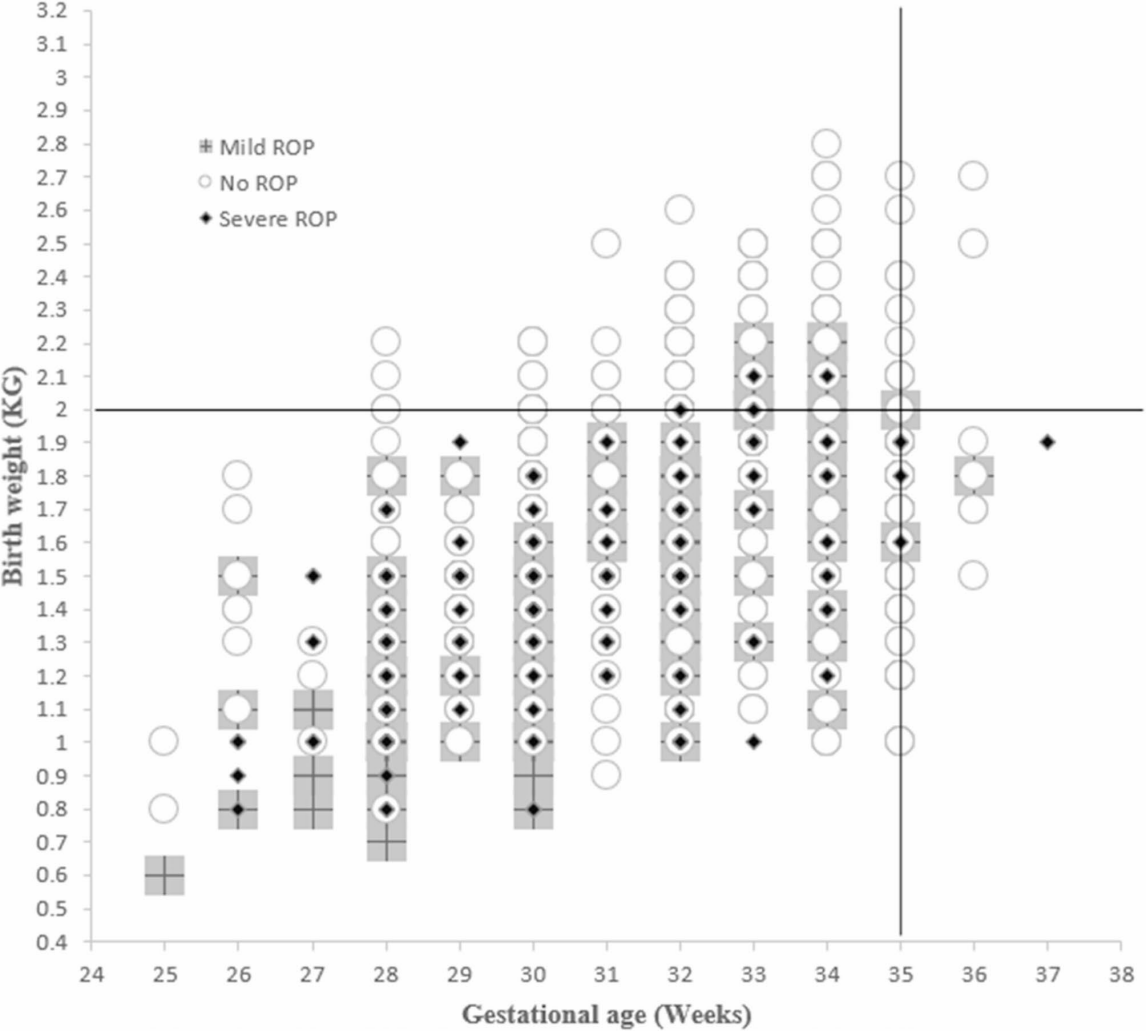
Scatterplot of gestational age and birthweight by ROP status. ^*^Data points could represent more than one infant

A scatterplot of birthweight by gestational age of all infants screened by ROP status shows that no infant with severe ROP fell outside both screening criteria (Fig. 1). One infant requiring treatment had a GA of 37 weeks but had a BW of < 2000 g; two infants had a BW of > 2000 g but both had a GA of < 35 weeks.

## Changes over time in the number screened and the findings of screening

**Fig. 2:**
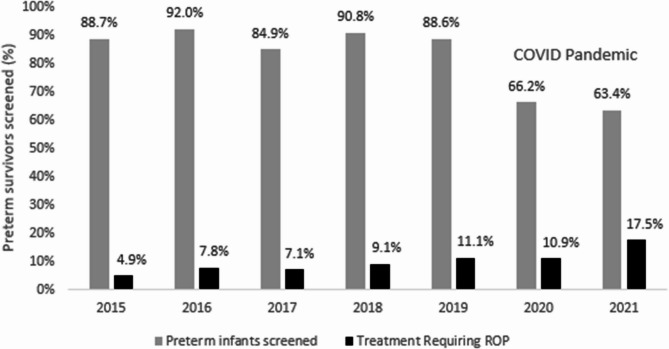
Proportion of surviving preterm infants screened and rates of Type 1 ROP before and during the COVID pandemic

The proportion of surviving eligible infants who were screened varied over time (Fig. 2). In 2018, 90.8% of eligible infants were screened; coverage was much lower during the COVID-19 pandemic in 2020 and 2021 (66.2% and 63.4%, respectively). During the pandemic, the proportion of infants with Type 1 ROP was higher than before the pandemic years (17.5% and 10.9%, respectively) Table 3. 

**Table 3:** Study cohort demographics between 2015–2021

	2015	2016	2017	2018	2019	2020*	2021^*^	Total
Eligible for screening	413	389	510	569	587	530	525	3523
Infants survived (%)	235 (56.9%)	224 (57.6%)	299 (58.6%)	327 (57.5%)	334 (56.9%)	294 (55.5%)	314 (50.8%)	2038 (57.8%)
Infants screened								
Number screened (% of infants survived)	206 (87.7%)	206 (92.0%)	254 (84.9%)	297 (90.8%)	296 (88.6%)	183 (62.2%)	199 (63.4%)	1641 (80.5%)
Mean GA (weeks)	31.9	31.7	31.5	31.5	31.4	32.2	31.9	31.7
Mean BW (g)	1660	1640	1570	1590	1600	1700	1680	1630
Infants requiring treatment								
Number (% of infants screened)	10 (4.9%)	16 (7.8%)	18 (7.1%)	27 (9.1%)	33 (11.1%)	20 (10.9%)	35 (17.5%)	159 (9.7%)
Mean GA (weeks)	29.7	30.5	29.6	29.9	29.8	31.7	30.8	30.3
Mean BW ROP (g)	1380	1480	1340	1360	1340	1480	1460	1400

^*^COVID Pandemic years

## Discussion

A recent systematic review and meta-analysis, which used the same definition of severe ROP as in our study (personal communication), estimated that the proportion of preterm infants screened globally who developed any ROP and severe ROP were 30% and 7%, respectively [17]. In our study, the incidence of any ROP and severe ROP were 32.2% and 9.7%, respectively which are very similar. The global data hide variation between countries. For example, severe ROP developed in 13.2–17.7% of infants in India and 26.3% of infants in Egypt [18–20]. but two of the these studies used a different definition of severe ROP [18, 19]. Lower rates of severe ROP were reported from private, level 3 NICUs in Pakistan (7.7% in 2015, 6% in 2013, 20.6% in 2008, and 3.2% in 2022) [11, 21, 22]. These institutions have enough oxygen blenders and pulse oximeters for every baby receiving supplemental oxygen, which are often not available in public hospitals like LGH. For example, in Mexico only 25.5% of cots had air-oxygen blenders and 80.1% had saturation monitors [6]. Two smaller one-year studies from a public hospital in Pakistan reported lower rates of severe ROP of 2.3% in 2016 and 5.8% in 2020 [12, 13]. Variability in rates of severe ROP is multifactorial and can be due to differences in the characteristics of the infants receiving neonatal care, the quality of neonatal care and survival rates, the definition of ROP and the screening criteria used, screening coverage and the completeness of screening, and the skill of the examining ophthalmologist. However, the relationship between the quality of neonatal care and the risk of ROP is not linear. Units providing poor-quality care may have low rates of ROP as the most at-risk infants do not survive. Units providing very high-quality care can also have low rates of severe ROP as modifiable risk factors are well controlled. Low follow-up rates at public hospitals may also play a role on reported rates, as demonstrated by a study from Lahore, Pakistan, where only 63% of referred patients were followed up, suggesting that the sickest infants, who are at higher risk for severe ROP, may not survive long enough to be included in follow-up data [23]. 

In our study, we used the screening criteria of a GA of ≤ 35 weeks or a BW of ≤ 2000 g, a decision based on the best available evidence and clinical judgment [24]. The OSP formally adopted these criteria in 2019. In this study, if the criteria of GA ≤ 30 weeks or a BW of ≤ 1500 g had been used, only 111/199 (55.6%) of infants with any ROP and 116/170 (68.2%) infants with severe ROP would have been identified. In our study, three infants with severe ROP fell outside one criterion but fell within the other; most infants (156/170, 91.8%) fell within both criteria. These findings are consistent with other studies in Pakistan, which show that these wide criteria captured the majority of affected infants [25]. In addition, in our earlier study of 51 infants who were blind from untreated ROP, only two (4%) had a GA of > 35 weeks and one (2%) had a BW of > 2000 g, demonstrating the appropriateness of these initial screening guidelines for public hospitals in Pakistan [8]. 

As in other studies, more preterm, sicker infants required more examinations and had longer inpatient stays than more mature infants [26]. A challenge of short inpatient stays is that most ROP screening needs to be conducted as an outpatient, which requires good counseling and communication with parents which needs to be considered at the planning stages. A lack of understanding of ROP among neonatologists in Pakistan can also make it more challenging to ensure proper follow-up. For example, in a survey of 62 neonatologists in Pakistan, 93% were aware of ROP, but only 69% believed infants should be examined for ROP and 32% were unsure of the treatment options [27]. It is also important to consider cultural factors; around a third of women in Pakistan give birth at home, and many change addresses as they move from their parent’s home to their husband’s house after delivery, which further complicates subsequent ROP follow-up examinations.

The lower attendance for screening at LGH during 2020 and 2021 can be attributed to the impact of the COVID-19 pandemic, which created additional barriers for families to access medical care. This trend was also observed in other LMICs, where the number of preterm infants admitted often fell, regular screening services could not be provided, and fear and lack of transport impeded adherence to screening after discharge from the neonatal unit [28]. Dedicated ROP coordinators can improve follow up rates, by collecting good data and providing essential counselling and support for families [29]. 

A limitation of this study is that it was undertaken on only one neonatal unit. Retinal imaging was not performed to verify the clinical signs and classification of ROP, but all infants were examined by ophthalmologists experienced in evaluating ROP.

Pakistan has a population of 240 million with high birth rates (27 per 1,000 population), and rates of preterm birth (14.4%).(35) Estimates for 2020 show that there were 921,600 preterm births (GA < 37 weeks) in Pakistan, the second highest of any country in the world [30]. We followed the methods used by Blencowe to estimate the annual incidence of Type 1 ROP and blindness from ROP in Pakistan [4]. Assuming that 15% of all preterm infants in Pakistan (921,600) are born at a GA of < 32 weeks (138,240 infants), 50% access neonatal care (69,120), 50% survive (34,560) [30, 31] and 16% of survivors develop Type 1 ROP, we anticipate that a minimum of 7,200 infants with a GA < 32 weeks will develop Type 1 ROP annually. The number will be higher than this because infants with a GA of 32–35 weeks can also develop Type 1 ROP. Without treatment approximately 15% of infants with Type 1 ROP would progress to irreversible blindness each year [16]. However, lack of data on the causes of blindness in children in Pakistan makes it difficult to ascertain and monitor the proportion due to ROP. Access to neonatal care and survival are likely to improve in the future, which will increase the screening and treatment burden. This highlights the urgent need for robust ROP screening and treatment programs not only in Pakistan but also in other countries with similar demographics.

This study can guide those caring for premature infants at risk of ROP to determine the likely disease burden and the resources needed to establish effective ROP services in Pakistan. In similar settings, for every 100 infants born at a GA of ≤ 35 weeks, 240 examinations would be needed, and 10 infants would require treatment for ROP. This information can aid in NICU resource planning and screening schedules and referrals. Re-evaluations every 6 to 12 months would improve burden assessment and resource allocation, ultimately enhancing patient care as ROP services expand.

## Conclusions

The third epidemic of ROP has arrived in Pakistan and demands immediate action through effective screening and treatment programs. As this study demonstrates, robust ROP screening programs can be established in public hospitals with some additional resources. This requires committed ophthalmologists and neonatologists, and a dedicated ROP coordinator. To avoid missing treatable disease in more mature preterm infants, the < 35 weeks screening criterion is essential. Outpatient screening and treatment will be necessary for most infants, emphasizing the need for comprehensive program planning. The findings of this study can guide institutions in estimating disease burden and allocating appropriate resources for ROP screening and treatment.

## Data Availability

The datasets used and/or analyzed during the current study are available from the corresponding author on reasonable request.

## Abbreviations

ROP: Retinopathy of prematurity
LMIC: low middle income country
HIC: high income country
GA: gestational age
BW: birthweight
OSP: Ophthalmological Society of Pakistan
IMR: infant mortality rate
LGH: Lahore General Hospital
PMA: postmenstrual age
